# Intra-Articular Administration of Autologous Purified Adipose Tissue Associated with Arthroscopy Ameliorates Knee Osteoarthritis Symptoms

**DOI:** 10.3390/jcm10102053

**Published:** 2021-05-11

**Authors:** Marco Caforio, Carmelo Nobile

**Affiliations:** Department of Pharmacy, Health and Nutritional Sciences, University of Calabria, Arcavacata di Rende, 87100 Cosenza, Italy; carmelo.nobile@unical.it

**Keywords:** osteoarthritis, knee, cartilage, regenerative medicine, adipose tissue, mesenchymal stem cells, Lipocell

## Abstract

The aim of this study was to evaluate the safety and efficacy of the intra-articular administration of autologous purified adipose tissue to treat knee osteoarthritis (OA) following arthroscopy. Thirty patients with radiological evidence of knee OA were recruited. A small liposuction and arthroscopic lavage and debridement were performed in the same surgical time. The harvested fat was processed intraoperatively with Lipocell (Tiss’You, RSM) to purify the adipose tissue injected into the knee. Clinical evaluations were performed with VAS, Womac, and Lequesne questionnaires before treatment and after 1, 3, 6, and 12 months of follow-up. Pain, measured with VAS, significantly decreased, showing a reduction of 53% after 1 month and 83% after a year. Functional recovery, measured with Womac, showed an improvement of 47% after 1 month post-treatment and 84% after 1 year. No adverse effects have been observed. The intra-articular administration of purified adipose tissue associated with arthroscopic lavage and debridement is a safe and significantly effective strategy in improving the symptoms of knee osteoarthritis in up to 1 year of follow-up.

## 1. Introduction

Osteoarthritis (OA) is a chronic degenerative disease with a high prevalence in the elderly population [1]. It is a joint disorder that causes cartilage degeneration, which affects all joints, especially the knees. Its symptoms include acute pain, limited mobility, and joint swelling. Conservative treatments and pharmacological solutions may help in the early stages of the disease, but the loss of cartilage progressively worsens the clinical picture [2]. Infiltration treatments with corticosteroids [3], hyaluronic acid [4], or injections of platelet-rich plasma (PRP) [5] only resolve pain management, and there is no consensus on their effectiveness [6]. Total knee replacement is considered the only possible solution to treat OA pain, but it is an irreversible and invasive surgery. With a longer life expectancy, patients could currently face two or more joint replacement surgeries in a lifetime, increasing possible complications [7]. Additionally, obese patients have higher risks, so they are not good candidates for a prosthetic implant. 

Regenerative medicine is a hot topic in orthopedics. Adult stem cells, e.g., mesenchymal stem cells (MSCs), can help tissue regeneration through cell differentiation and cytokine release [8]. MSCs are found in many tissues, but recently adipose tissue has been put under the spotlight. The abundance in MSCs frequency compared with other sources such as bone marrow [9] make adipose tissue an interesting stem cell source.

In vitro and animal studies show that adipose-derived MSCs (AD-MSCs) can promote cartilage repair by exerting a paracrine effect with the release of trophic and anti-inflammatory molecules [10]. Clinical trials demonstrated that the local injection of autologous AD-MSCs is safe and well-tolerated in OA patients [11]. Notably, lower doses of AD-MSCs correlate with better improvements suggesting a dose-independent therapeutic effect [12]. One single cell line can serve for the preparation of thousands of precise AD-MSCs doses in vials. In the future, these could be delivered by practitioners without even performing lipoaspiration on patients, if regulatory approval is obtained. While waiting for more extensive studies to confirm these preliminary results, the scientific community explored the possibility of exploiting the regenerative potential of AD-MSCs with minimally-manipulated adipose tissue derivatives. Point-of-care medical devices help break and purify adipose tissue without MSC isolation [13]. This option is more convenient in orthopedic daily practice for the current American and European regulation requirements on cell and tissue therapies. Many publications demonstrate the safety of adipose tissue derivatives, with significant improvement of OA symptoms [14,15,16,17,18], as a valid alternative to cell expansion and enzymatic digestion of adipose tissue [19].

Arthroscopic lavage and debridement is commonly performed when initial conservative measures fail to evaluate joint balance and ameliorate joint homeostasis, but long-term efficacy is poor [20,21] with no significant benefit in comparison to sham surgery [22]. For this reason, our study explored the association of benefits of both arthroscopic lavage and the delivery of AD-MSCs. Here we present the clinical follow-up of a cohort of 30 OA patients treated with intra-articular injection of purified lipoaspirate obtained intra-operatively with Lipocell, a class-IIa medical device, following arthroscopy.

## 2. Materials and Methods

### 2.1. Patient Recruitment

This study was approved by the Ethics Committee of Università della Calabria (Prot. n. 11199, 14 May 2016). From 2018 to 2019, 30 patients (Table 1) with knee pain were recruited. Selection criteria were radiological evidence of knee OA according to the Kellgren-Lawrence scale and BMI between 18 and 31 kg/m^2^. Exclusion criteria were axial deformity, ligament instability, severe meniscal tears, e.g., complete ruptures or bucket-handle tears, algodystrophy, synovitis, rheumatoid arthritis, kissing lesions, and also corticosteroids or hyaluronic acid injections in the past 3 months. All patients signed informed consent and filed VAS, Womac, and Lequesne questionnaires before treatment.

### 2.2. Sample Collection and Processing

After spinal anesthesia, a knee arthroscopic lavage and debridement was carried out on all patients. The arthroscopy involved the use of chondral shaver to clean jagged fibrous tissue with motorized aspiration of all debris. Then, the abdominal subcutaneous area was infiltrated with Klein solution (lidocaine 2% and adrenaline 1 mg/mL in 500 mL NaCl 0.9% solution) to perform small liposuction (~50 mL). Fenestrated blunt cannulae were used to collect lipoaspirated fat from abdominal subcutaneous fat. Subsequently, lipoaspirates were processed with Lipocell (Tiss’You, RSM) [13]. Following the instructions, the lipoaspirate was inserted into the device for filter dialysis and Ringer Lactate washing (300–500 mL). The filter consists of a 50 µm membrane that retains the lipoaspirate and allows it to be washed of blood and oil residues. After 3–5 min, the lipoaspirate appeared clean of the blood, and the washing solution was also transparent in the outflow tube; therefore, the lipoaspirate was recovered with a 10 mL syringe from the outlet connection.

### 2.3. Treatment and Follow-Up Protocol

Six milliliters of purified adipose tissue was injected into the knee with an 18G needle at the end of the knee arthroscopy, after removing the water. Hence, the knee was immobilized in flexion and extension to help spread the product within the joint. Ice packs (3 days) and compressive dressing (10 days) were recommended for harvested and treated areas. The knees were partially unloaded in the first 5 days with the help of crutches. Then, only one crutch was kept on the contralateral side for another 5 days. The mobilization took place immediately. The return to hard work and sports activity was postponed for 30 days after the treatment.

### 2.4. Clinical Evaluation and Statistical Analysis

Patients were examined with the Womac, Lequesne, and VAS questionnaire at 1, 3, 6, and 12 months after the treatment. Patients were also observed for adverse effects, e.g., synovial reactions. Mild knee swelling caused by the infiltration and abdominal ecchymosis caused by the liposuction were expected. All data differences with pre-treatment values were tested with non-parametric Wilcoxon tests for statistical significance.

## 3. Results

All patients tolerated the treatment well, and no severe adverse effects were observed, except small and temporary ecchymosis on harvested areas and mild and temporary knee swelling. Clinical questionnaires showed a significant improvement both in pain relief and functional recovery 1 month after the treatment and even better improvement in longer follow-up, such as 1 year after the procedure.

**Figure 1:**
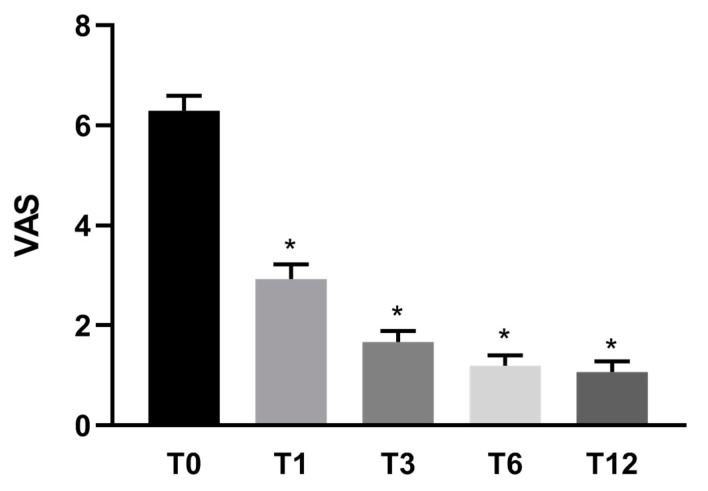
Mean VAS score (*n* = 30) before and after Lipocell treatment. T0 is pre-treatment, Tx is x months after the treatment. Error bars show SEM * *p* vs. pre-treatment <0.01.

**Figure 2:**
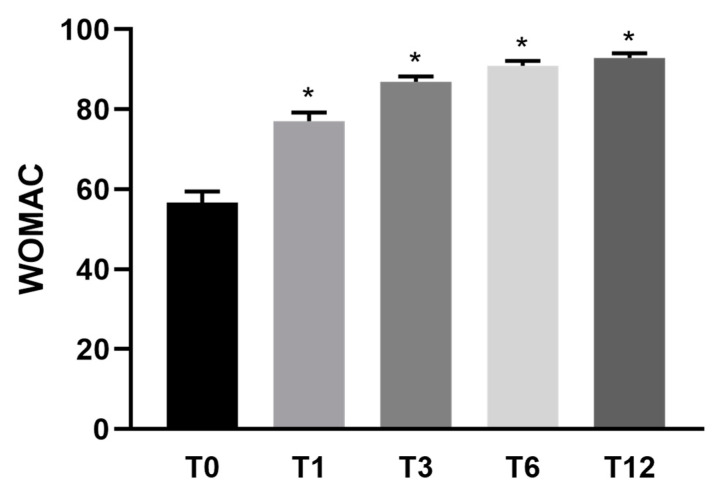
Mean WOMAC functional score (*n* = 30) before and after Lipocell treatment. T0 is pre-treatment, Tx is x months after the treatment. Error bars show SEM * *p* vs. pre-treatment <0.01.

**Figure 3:**
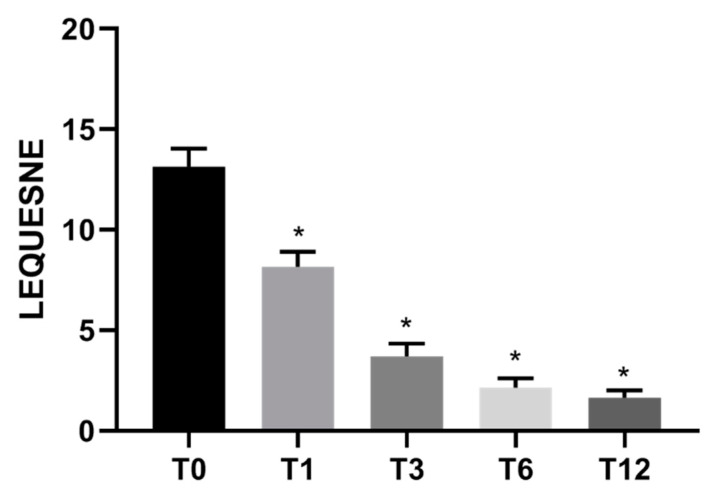
Mean Lequesne functional score (*n* = 30) before and after Lipocell treatment. T0 is pre-treatment, Tx is x months after the treatment. Error bars show SEM * *p* vs. pre-treatment <0.01.

Specifically, pain measured with VAS showed a reduction of 53% (±3) after 1 month, 74% (±2) after 3 months, 81% (±2) after 6 months, and 83% (±2) after 1 year in comparison to pre-treatment conditions (Figure 1). Knee functional recovery assessing pain, rigidity, and quality of life (Womac questionnaire) showed an improvement of 47% (±6) after 1 month, 70% (±3) after 3 months, 79% (±2) after 6 months, and 84% (±2) after 1 year in comparison to pre-treatment conditions (Figure 2). Finally, the Lequesne questionnaire assessing knee disability showed a trend of improvement similar to Womac results (Figure 3). 

## 4. Discussion

The intra-articular administration of autologous purified adipose tissue has been discovered to be a safe and effective treatment for the improvement of knee OA symptoms. Numerous studies have confirmed the results of this procedure, in which simple intra-articular infiltrations of purified autologous adipose tissue were performed [14,15,17,22]. Our research suggests that this treatment can also improve the arthroscopic lavage and debridement outcome. Notably, arthroscopy is commonly used in degenerative knees that failed conservative management, but the long-term results are poor [20,21], with no more benefit than sham surgery [23]. Because arthroscopic evaluation is helpful to evaluate the status of the joint, lavage and debridement can be recommended to set up an ideal joint environment for subsequent cell therapies by removing debris and lowering inflammation. Indeed, preliminary reports on the safety of intra-articular administration of fragmented adipose tissue associated with arthroscopy showed no cases of post-arthroscopic femoral condyle necrosis or algodystrophy [24].

AD-MSCs exert an immuno-modulatory function on synovial membranes by releasing anti-inflammatory cytokines [8]. Moreover, in vitro studies show that AD-MSCs can release specific growth factors to stimulate cartilage remodeling [10]. Articular cartilage regeneration remains a challenge, and many recent strategies for OA treatment are based on it [22]. Our study could not demonstrate or claim cartilage regeneration or remodeling with histological analysis due to the invasiveness of biopsies; still, all the patients reported satisfactory results after the treatment. Furthermore, our study did not show possible radiological changes in OA progression, although we did not expect any, as observed in comparable studies [17]. In longer follow-ups, X-ray imaging may help to show a slowing effect in OA progression.

A limitation of this study was the lack of a control group. Therefore, we could not rule out the presence of a placebo effect, which can be crucial for pain symptoms [5,25]. Indeed, the presence of a control group is essential to confirm the efficacy of the described treatment versus arthroscopy alone, and will be a subject of our next study; moreover, further investigations will compare this new treatment with standard options such as hyaluronic acid or PRP injections.

Some patients were old enough to be candidates for joint replacement. However, in daily clinical practice, there is strong demand for conservative treatments, and cellular therapies can offer an ideal solution. Therapeutic tolerance was high, and no complications were observed. There was some swelling on the treated knees, and the only recommendation was to postpone the removal of the compression dressing. At 1 month, none of the patients reported swelling or other adverse effects.

Although some studies show that AD-MSCs can be isolated in vitro and injected with satisfactory clinical results [11,12], EU and American regulations impose specific requirements when using cell and tissue therapies. In Italy, the treatment of osteoarthritis using minimally manipulated adipose tissue derivatives is approved by the Centro Nazionale Trapianti (July 2015). Neither the European Medicines Agency nor Istituto Superiore della Sanità have made any definitive statements on this topic. In our study, we used a class-IIa medical device that is CE-approved and meets the minimum handling requirements [13], resulting in an optimal, cheap, and reproducible approach. Overall, the results of this new therapy have been satisfactory; however, further studies are needed to observe the effects of longer follow-ups and on a larger population.

## Figures and Tables

**Table 1:** Demographic data

Patient (*n*)	30
Age (year)	60 (range 36–77)
Gender (*n*)	
Male	17
Female	13
Kellegren-Lawrence (*n*)	
II	14
III	13
IV	3

## Data Availability

Data available on request due to privacy restrictions.
